# To determine the frequency of stroke and common factors leading to it after coronary artery bypass grafting

**DOI:** 10.12669/pjms.37.1.3242

**Published:** 2021

**Authors:** Sayed Mumtaz Anwar Shah, Mujeeb Ur Rehman, Nabil I Awan, Azam Jan

**Affiliations:** 1 Dr. Sayed Mumtaz Anwar Shah, FCPS. Assistant Professor, Department of Cardiothoracic Surgery, Rehman Medical Institute, Peshawar, Pakistan; 2 Dr. Mujeeb Ur Rehman, MS. Senior Medical Officer, Department of Cardiothoracic Surgery, Rehman Medical Institute, Peshawar, Pakistan; 3 Dr. Nabil I Awan MBBS. Post-Graduate Resident, Department of Cardiothoracic Surgery, Rehman Medical Institute, Peshawar, Pakistan; 4 Dr. Azam Jan, ABTS, Head of Department, Department of Cardiothoracic Surgery, Rehman Medical Institute, Peshawar, Pakistan

**Keywords:** Stroke, diabetes mellitus, atrial fibrillation, myocardial infarction, aging, atherosclerosis, CABG, epidemiology, risk factors

## Abstract

**Objective:**

To determine the frequency of stroke and common factors leading to it after coronary artery bypass grafting.

**Methods:**

This study was conducted at Cardiothoracic Surgery Department, Rehman Medical Institute, Peshawar. Study design was descriptive cross sectional study and the duration of the study was six months. The total sample size was 183 cases using 8.3% frequency of stroke after coronary artery bypass grafting, 95% confidence interval, 4% margin of error, using WHO sample size calculator.

**Results:**

In this study mean age was 45 years with SD ± 1.26. Seventy five percent patients were male while 46(25%) females. Six patients (3%) patients had stroke while 177(97%) patients did not have any stroke. Among the six patients who were analyzed, all of them had Diabetes Mellitus and 50% patients had recent AMI, while only one patient had prior history of Atrial Fibrillation.

**Conclusions:**

Diabetes Mellitus is an independent risk factor for stroke after CABG surgery. whereas, recent MI is also associated with increased incidence of stroke in post CABG patients.

**Abbreviations**
OPCAB: off-pump coronary artery bypass,
CABG: Coronary artery bypass grafting surgery,
POAF: Postoperative atrial fibrillation,
MI: myocardial infarction,
LMS: Left main stem,
CPB: cardiopulmonary bypass,
AWMI: anterior wall myocardial infarction,
AMI: Acute myocardial Infarction.

## INTRODUCTION

Despite advances in anesthesia and cardiac surgery techniques, such as the use of off-pump coronary artery bypass (OPCAB), stroke remains the most devastating neurological complication of surgical myocardial revascularization with an established increased risk of morbidity and mortality.^1^

It is reported that on an annual basis, an approximate 42000 patients worldwide and 21000 patients in the US suffer from stroke after cardiopulmonary bypass. This in effect, has increased the annual health care cost from two billion to four billion dollars presumably due to a longer in-hospital stay and the need for prolonged rehabilitation services.^2^

The incidence of stroke after coronary artery bypass grafting surgery (CABG) in our country has been reported to be between 1.3 and 4.3%.^3^ Similarly, John Hopkins Hospital maintains a stroke database and in that they have cited a per-operative stroke rate of 4.5% in patients undergoing CABG surgery.^4^

A significant proportion of these patients will exhibit adverse reversible or permanent neuro psychological complications making post-CABG stroke a very serious health issue. In addition, patients having postoperative stroke have been identified to be at an increased risk of early and late mortality. Therefore, it is imperative to be able to identify the pre-operative risk factors associated with post-operative stroke.

A number of pre- and per-operative factors can predict the likelihood of post-cardiac surgery stroke. A history of diabetes, hypertension, increased age, atrial fibrillation, preoperative neurological event, aortic atheromatous / calcific disease, bilateral carotid artery disease, aortic cross clamp time or evidence of mural thrombi are all related with increased risk of neurological damage after CABG.^5^ In a study conducted by Oliviera DC et al; the number of re vascularized vessels was demonstrated to be directly proportional to the occurrence of stroke after CABG. In addition, it was observed that systemic hypertension and diabetes mellitus were both independent predictors of post-operative stroke in CABG patient within the first 24 hours.^6^

At the National University Hospital in Singapore, 2.7% patients were found to have suffered from neurological complications post-cardiac surgery. Similarly, in another study a 3% incidence of stroke was observed in the 688 patients who underwent cardiac surgery.^7^ In Pakistan, very limited data is available about the incidence of stroke in CABG patients or the risk factors that are commonly found. The aim of this study was to determine the frequency of stroke after coronary artery bypass grafting in addition to getting an idea of the common risk factors that are found to be associated with it.

## METHODS

This descriptive cross sectional study was conducted at Cardiothoracic Surgery Department, Rehman Medical Institute, Peshawar from October 10, 2019 to April 25, 2020.

### Sample size

Total 183 cases using 8.3%^4^ frequency of stroke after coronary artery bypass grafting, 95% confidence interval, 4% margin of error, using WHO sample size calculator.

### Sampling technique

Non-probability consecutive sampling

### Inclusion criteria:

- All patients with coronary artery disease (double and triple vessel disease and left main stem disease) undergoing coronary artery bypass grafting.
- Both male and female patients.
- Patients of age 60 years and below.

### Exclusion criteria:

- Chronic obstructive pulmonary disease patients as diagnosed by FEV_1_/FVC ratio less than 70% of predicted value.
- Chronic renal failure as diagnosed by pre-operative serum creatinine level above 200 µmol/l.
- Rheumatic heart disease detected by echocardiography.

The above mentioned conditions act as confounders and if included would have introduced bias in the study results.

### Data collection procedure

The study was conducted after approval from the research and ethical committee of our hospital with Ref No: RMI/RMI-REC/Article Approval/34, June 22, 2020. All patients meeting the inclusion criteria (patients with double vessel, triple vessel disease or left main stem disease diagnosed by coronary angiography) were enrolled in the study through OPD and subsequently admitted in the cardiovascular surgery department of the hospital for further work up. Routine On-Pump CABG procedure was done for all the patients.

The purpose and benefits of the study were explained to the patient and a written informed consent was obtained. All patients were worked up with a detailed history and clinical examination followed by routine pre-operative investigations. All the patients were put on next OT list for coronary artery bypass grafting as per ward protocols.

Routine blood investigations along with chest X-ray, Echocardiography, Electrocardiography, Angiography, Carotid Doppler and Pulmonary Function Tests were done pre-operatively for all the patients. Carotid Doppler was indicated in all symptomatic and asymptomatic carotid artery disease patients. Pulmonary Function Tests was indicated in old, smoker and other lung disease patients.

### Definitions:

### 1. Carotid artery disease:

Indicate whether the patient ever had a non-invasive/invasive carotid test with > 75% occlusion. This test is also known as a carotid Doppler study. An angiogram of the carotid arteries can also be performed by magnetic resonance angiography (MRA).

### 2. Prolonged cross clamp time:

Indicate the total number of minutes the aorta is completely crossed-clamped during bypass. Minutes should not be recorded if partial cross clamp is the highest level of occlusion. Aortic cross clamp time >100 minutes was considered as prolong cross clamp time.

### 3. Recent myocardial infarction:

MI within 7-30 days before the procedure.

### 4. Prior atrial fibrillation:

the electrical signals that coordinate the muscle of the upper chambers (atria) of the heart become rapid and disorganized; resulting in an irregular heartbeat (arrhythmia) often greater than 300 beats per minute.

### 5. Diabetes mellitus:

Capture the presence and or history of diabetes mellitus, regardless of duration of disease or need for anti-diabetic agents diagnosed prior to surgical intervention. A diagnosis is typically made from a series of blood glucose levels or other diagnostic tests and not a onetime elevated value. DO NOT include gestational diabetes or the term borderline diabetes. The risk factor of diabetes should be documented upon admission or as a preoperative diagnosis.

All patients underwent standard coronary artery bypass grafting by the same consultant cardiovascular surgeon who had more than seven years of surgical experience. Postoperatively, all patients were observed daily till 6^th^ post op day for any signs of neurological deficit. Once detected, common factors leading to stroke were scrutinized i.e. Recent AMI, significant carotid artery disease, Diabetes Mellitus, prolonged aortic cross clamp time and past history of atrial fibrillation.

All the above mentioned information including name, age, and gender was recorded in a pre-designed pro forma. An exclusion criterion was strictly followed to control confounders and bias in the study results.

### Data analysis procedure

All data was stored and analyzed on SPPS version 14. Mean ± SD was calculated for quantitative variables like age. Frequencies and percentages were calculated for categorical variables like gender, age, stroke and the common factors that lead to it, Recent AMI, significant carotid artery disease, Diabetes Mellitus, prolonged aortic cross clamp time and Past history of atrial fibrillation). Stroke and common factors leading to stroke were stratified according to age and gender to see the effect modifications. All results were presented in the form of tables and graphs.

## RESULTS

This study was conducted at Cardiovascular & Thoracic Surgery Department of our hospital. Study design was a descriptive cross sectional study and the duration of study was six months. A total of 183 patients were observed to determine the frequency of stroke and the common factors leading to it after coronary artery bypass grafting and the results were analyzed as. We have used the SPSS and Chi-square test for analysis in which the P-value less than 0.05 is statistically significant.

Age distribution among 183 patients was analyzed. It was found that 46(25%) patients were in the age range of 38-44 years, 68(37%) patients were in the age range 45-50 years, 51(28%) patients were in the age range 51-55 years, and 18 (10%) patients were in the age range of 56-60 years. Mean age was 45 years with SD ± 1.26. Gender distribution among 183 patients showed 137(75%) males and 46(25%) females (Table-I). the analysis has been done by using SPSS.

**Table-I:** Frequency of common risk factors in stroke.

Common Risk Factors	Frequency	Percentage
Recent AMI	3	50%
Significant Carotid Artery Disease	Nil	Nil
Diabetes Mellitus	6	100%
Prolonged Aortic Cross Clamp Time	Nil	Nil
Prior history of Atrial Fibrillation	1	16%
Total	6	100%

Out of the 183 patients who were analyzed, 6(3%) patients had stroke while 177(97%) patients did not have a stroke and amongst the 6 patients who had stroke, it was found that all the six patients had Diabetes Mellitus, 3(50%) patients had recent AMI and 1(16%) had prior history of atrial fibrillation. Table-II.

**Table-II:** Age Distribution.

Age	TOTAL(n=183)	Stroke	No Stroke	P-Value
	38-44 years	46(25)	0(0)	46(25.9)	0.000
	45-50 years	68(37)	1(16.67)	67(37.85)	0.001
	51-55 years	51(28)	3(50)	48(27.1)	0.003
	56-60 years	18(10)	2(33.33)	16(9)	0.002
Gender	Male	137(75)	4(2.9)	133(97.1)	0.003
	Female	46(25)	2(4.34)	44(95.66)	0.003

Mean age was 45 years with SD ± 1.26 Chi square test was applied for each P value

Stratification of common risk factors of stroke was done according to age distribution which showed that out of the 3 patients with recent AMI, one patient was in age range 38-44 years, one patient was in age range 45-50 years and one patient was in age range 51-55 years. In the 6 patients with Diabetes Mellitus, 2 patients were in age range 38-44 years, 3 patients were in age range 45-50 years and one patient was in age range 51-55 years. The one patient with prior history of atrial fibrillation was in age range 56-60 years (Table-III).

**Table-III:** Stratification of common risk factors in stroke with age distribution.

*Risk Factor*	*Frequency in Stroke*				

	38-44 years	45-50 years	51-55 years	56-60 years	Total

Recent Awmi	1	1	1	0	3
Diabetes Mellitus	2	3	1	0	6
Prior History of Atrial Fibrillation	0	0	0	1	1

Chi square test was applied in which P value was 0.063

Stratification of common risk factors of stroke was then done with Chi-square test according to gender distribution which revealed that the six patients with diabetes four patients were male and two patients were female. In three patients with recent AMI, 2 patients were male and one patient was female and one patient with prior history of atrial fibrillation was male (Table-IV).

**Table-IV:** Stratification of common risk factors in stroke with gender distribution.

*Risk Factor*	*Frequency in Stroke*			*P-value*

	Male	Female	Total	

Recent Awmi	2	1	3	<0.05
D.M	4	2	6	
Prior History of Atrial Fibrillation	1	0	1	

Chi square test was applied in which P value was <0.05;DM: Diabetes Mellitus; AWMI: Anterior Wall Myocardial Infarction;

## DISCUSSION

CABG is the most commonly performed major cardiac surgical procedure. The established rate of stroke in patient undergoing coronary artery bypass grafting has been cited by the Society of Thoracic Surgeons to be 1.6%.^8^

The mortality amongst these patients who have had a stroke after CABG has been reported to be around 20%.^9^ The perioperative risk of stroke in CABG patients is known to be related to certain factors that include hypertension, type II diabetes mellitus, older age, previous stroke, left main stem disease(LMS), smoking, mild renal impairment and female gender.^10^

Initially, the introduction of the off-pump CABG aimed to reduce the incidence of post-operative stroke and in 2009, a single-center study comprising of 2516 patients reported a decreased incidence of stroke in off-pump CABG patients. However, they could not demonstrate any significant difference in the frequency of delayed stroke when compared to on-pump CABG patients.^11^

Recently, the CORONARY trial, which is the largest RCT till date that compares five year outcomes of ONCAB versus OPCAB cited no significant difference in the incidence of post-operative stroke in the two groups.^12^ Therefore in our study we only included those patients who underwent on-pump CABG. We found the incidence of stroke to be 3% which is in the range that has been published by other centers in our demographic. The incidence of stroke in CABG patients has been locally reported 1.94% which is very low.^13^ The lower incidence of stroke has been reported in Off-pump Coronary Artery Bypass (OPCAB) patients as well as in single clamp group patients compared to multiple clamp patients.^13^ We applied single clamp technique for all the patients in our study but done On-pump CABG for all the patients. We expected that OPCAB procedure would decrease the incidence of stroke while combining with single clamp technique.

Local study has reported that Female sex was an independent risk factor for stroke and all the patents in stroke group were male.^14^ We reported the frequency of stroke in male patients 75%. In our study, the incidence of stroke was more in patients of age 51-55 years and the local study has reported the incidence in less than 60 years of age patients.^14^

Whether Diabetes Mellitus is a predictor/risk factor or not for post-operative stroke in patients undergoing CABG surgery has been investigated in quite detail. However, no consensus has been reached with multiple studies providing evidence on either side. In our study, we found that all the patients who had stroke were diabetics. Diabetes as an independent risk factor for stroke in the general population has been known for long however, further studies are needed to absolutely classify diabetes as a risk factor for stroke after CABG surgery.^15^-^19^

Post-operative atrial fibrillation is one of the most common complications after coronary artery bypass grafting (CABG), Postoperative atrial fibrillation (POAF) is reported to occur in 10% to 65% of all patients undergoing cardiac surgery and 25-40% of all patients undergoing CABG surgery.^20^,^21^ It has been an established fact now that patients who have post-operative AF report an increased risk of stroke. Our findings also echoed this as 16% of patients with stroke were found to have new onset atrial fibrillation.^22^

It has been reported in recent studies that the risk of stroke was higher not only after early CABG in recently occurred myocardial infarction (MI) but also when CABG was performed later. Nevertheless, the incidence of stroke was significantly higher after early CABG than if CABG was performed later.^23^ we learned that 50% of the patients who had stroke in our study had a recent MI. A local study done in 2016 showed that from amongst patients who had stroke following CABG surgery, all of them had a prolonged cross clamp time of more than >100minutes.^14^

It has been reported by multiple other researches that a cardiopulmonary bypass (CPB) time of more than 110 minutes is an independent risk factor for stroke. However, in our study none of the patients with stroke had a prolonged bypass time. This may be due to a very small proportion of our cohort having a prolonged CPB time as a whole and therefore little representation of that particular population in our results.^24^,^25^

One local study has reported the frequency of stroke 15% in significant critical carotid stenosis (>70% lesion) but the frequency of stroke was not significant in our study. Although there were patients with less than 50% carotid artery stenosis and we consider insignificant if the stenosis is less than 50% as we had ruled out by carotid doppler.^26^ The incidence has been reported 10.40 % in local study.^27^

### Limitations of the study

The current study has very clearly demonstrated that the incidence of stroke in CABG surgery is 3% and that diabetes mellitus, recent Acute Myocardial infarction, and Post-operative Atrial Fibrillation are present in patients that have a stroke post-CABG. However, risk predictive scores would have given more accurately information on the extent of risk associated with the above mentioned predictors. In addition, we studied the data from patients who had a stroke. This data should be compared with CABG patients who did not have a stroke. Lastly, in our study no significant effect of prolonged CPB time or significant carotid artery disease could be drawn on the incidence of post-operative stroke even though both these are established risk factors for stroke. The reason behind this is our small study population. More patients need to be added to this data to give us a clearer picture of the predictors of stroke.

## CONCLUSIONS

We found that Diabetes Mellitus, recent Acute Myocardial Infarction and Post-operative Atrial Fibrillation are associated with stroke in patients undergoing Coronary Artery Bypass Grafting.

### Authors’ Contribution:

**NIA & MUR:** Conceived, designed and did statistical analysis & manuscript writing.

**SMAS:** Did data collection and editing of manuscript.

**SMAS & AJ:** Did review and final approval of manuscript.

**MUR** will be responsible and accountable for the accuracy or integrity of the work.
